# Innate Lymphoid Cells in Crohn’s Disease

**DOI:** 10.3389/fimmu.2020.554880

**Published:** 2020-11-16

**Authors:** Ying Wu, Jun Shen

**Affiliations:** Division of Gastroenterology and Hepatology, Key Laboratory of Gastroenterology and Hepatology, Ministry of Health, Inflammatory Bowel Disease Research Center, Renji Hospital, School of Medicine, Shanghai Institute of Digestive Disease, Shanghai Jiao Tong University, Shanghai, China

**Keywords:** innate lymphoid cells, Crohn’s disease, mucosal homeostasis, innate immune system, inflamma

## Abstract

Innate lymphoid cells (ILCs) are a large family of cells of the immune system that performs various functions in immune defense, inflammation, and tissue remodeling. As a part of the innate immune system, ILCs are a distinct form of lymphocytes different from T and B cells. ILCs can provide host defense against the source of infection and initiate the repair and remodeling processes to restore and maintain host body homeostasis. The number of patients with Crohn’s disease (CD) worldwide has continued to increase in recent years and this disease has brought sickness and death to many families. Numerous studies have found that ILCs also undergo a series of alternations during the development of CD and contribute to this disease. Despite this, the pathogenesis of CD is still not fully explained. So, we keep researching and exploring. In this review, we have closely linked the latest progress on ILCs and CD, and introduced, in detail, the specific roles of four different types of ILCs in CD. We also describe new progress in the pathogenesis of CD, with particular emphasis on the plasticity of ILC3s in this disease. These new studies and findings may provide new insights and breakthrough points for the treatment of CD.

## Introduction

Innate lymphoid cells (ILCs) originate from common lymphoid progenitor cells (1). Although similar to T cells, they do not express antigen-specific T- or B-cell receptors. ILCs distribute throughout the organs are enriched on the mucosal surface (2). ILC precursors develop into natural killer (NK) cells and a series of ILC subsets. Among them, NK cells are the representatives of the cytotoxic ILC population and differ from noncytotoxic ILCs in cytotoxicity and the ability to be activated in response to viral infections and tumors. Classification of helper ILCs is based on their specific expression of key transcription factors or cytokines. They can be divided into three subsets: type 1 ILC, type 2 ILC, and type 3 ILC (3, 4). Moreover, the expression patterns of cytokine receptors are also differ; type1ILCs express IL-12R-β2, type 2 ILCs express IL-33R and IL-25R, and type 3 ILCs express IL-1R and IL-23R (5).

**Figure 1:**
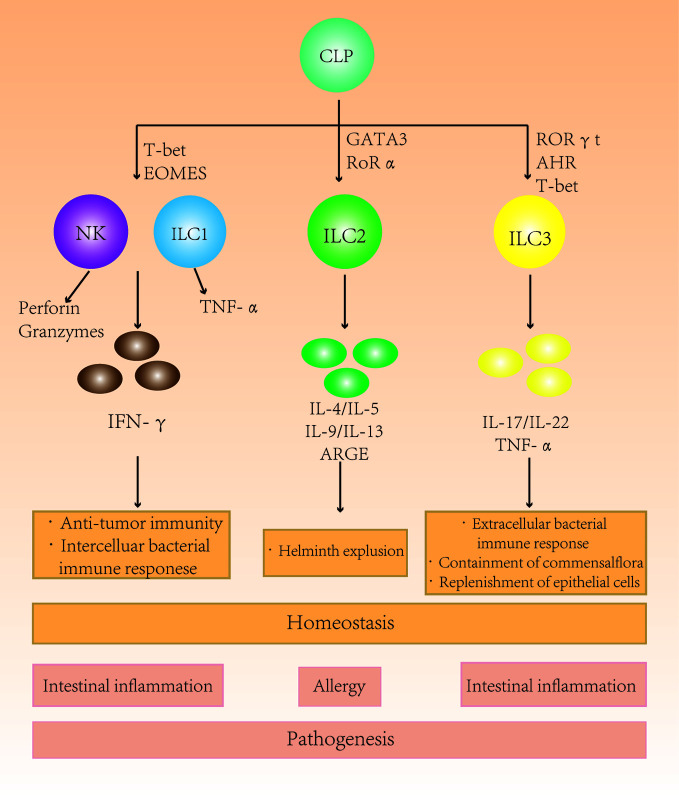
The role of different subgroups of innate lymphoid cells (ILCs) in the intestine. Different ILC subgroups secrete different characteristic cytokines that participate in intestinal immunity and maintain homeostasis. Changes in the ILC subtype ratio and subsequent secretion imbalances in the intestine can cause intestinal diseases.

Type 1 ILCs are enriched in the liver, skin, salivary glands, uterus, thymus, and bowel. In those organs, they perform a crucial role in enhancing immunity against intracellular bacteria and parasites. Type 2 ILCs are a subgroup of innate immune cells with the ability to secrete proinflammatorycytokines. They are GATA3^+^-interleukin (IL)-5 or IL-13–producing cells. The primary function of type 2 ILCs is to promote type 2 inflammation in allergies, invermination and tissue repair (6). The interaction between epithelium and type 2 ILCs mediates immunity to helminthic parasites. This type 2 immune response is characterized by production of a series of cytokines, including IL-4, IL-5, IL-9, and IL-13 (7). Type 3 ILCs exist mainly in mucosal tissue and at low levels in spleen and liver. IL-22 selectively acts on stromal cells and epithelial cells, leading to the rapid production of antibacterial peptides and has excellent effects on preventing transmission. Type 3 ILCs are RAR-related orphan receptor gamma (RORγ) t^+^-IL-17A– or IL-22–producing subset (5). They can drive immune tolerance in the host gut by controlling the function of other immune cells (**Figure 1**). For example, type 3 ILCs can control the activation of auto-reactive CD4^+^ T cells in the lamina propria through major hisoconpatibility complex-II (MHCII) expression. MHCII-expressing CCR6^+^type 3 ILCs were found to control intestinal homeostasis through induction of apoptotic cell death and deletion of activation commensal bacteria-specific T cells (8, 9). MHCII^+^type 3 ILC may represent a novel therapeutic target to control pathologic CD4^+^T cell responses in chronic inflammatory disorder associated with dysregulated host-commensal bacteria relationships (10, 11).

Microbiota is closely related to ILC development. ILCs and the gut microbiata communicate with each other in an indirect manner via cytokine signaling, and these signals also combine with signals from intestinal epithelial cells (IECs) and macrophages (12). The commensal flora of the gut and the skin is known to directly influence the development of certain lymphocyte populations (13–15). The host immune system, in turn, shapes the structure and function of the gut microbiota (16, 17). ILCs react to the gut microbiota by changing their structure, having protective or destructive effects on gut immunity. Among ILCs, the most important with regard to the gut microbiota are type 3 ILCs (12). The studies reported that intestinal type 3 ILC frequencies were severely reduced in mice lacking a commensal flora and frequencies increased with age in newborn mice with a few NKp46^+^ type 3 ILC in the intestine and thus correlating with commensal colonization (18–20). The signals from commensal flora affect the maturation of ILCs and the acquisition of tissue-specific functions of ILCs. Other studies (20, 21) indicated that commensal flora is not required for type 3 ILCs development may be due to the presence of certain resident microbiota that contribute to type 3 ILC development and function through the production of metabolites that activate Ahr (15, 22, 23).

ILCs have recently been shown to be connected to the enteric nervous system (ENS). Novel studies reported that when nerve cells receive an alert from the gut, specific instructions are passed on to ILCs to produce effectors that can repair intestinal damage. The process that protects the intestinal from inflammation and microbial infection via interactions between enteric glial cells (EGCs) (essential components of the ENS) and type 3 ILCs through neurotrophic factor signals (12, 24). IL-22 secretion is up-regulated by activated enteric type 3 ILC via ECGs or/and directly regulated in the downstream of STAT3 activation by neurotrophic factors (24).

CD is a chronic inflammatory disease of the human intestine. The cause so far is unclear. Growing evidence indicates that dysregulated mucosal immune responses of the commensal microbiota contribute to this process and the tissue damaging process. ILCs exhibit a degree of functional diversity and plasticity, and this can be demonstrated in the studies of intestinal diseases such as CD. The frequency of type 1 ILCs and type 3 ILCs in the intestinal tract is apparently higher in patients with CD than in normal controls (25). IFN-γ–producing type 1 ILCs are found in intestinal tissue, particularly in CD patients with inflammation (7). Moreover, CD shows increased gene expression of vital type 3 ILCs cytokines (IL12A and IL22), transcription factors (RORC and AHR), and a cytokine receptor (IL23R). These findings suggest that type 1 ILCs and type 3 ILCs are closely related to the pathogenesis of CD (25, 26).

In a recent observational study of CD patients, Li et al. (27) mentioned that the frequency of Lineage^−^CRTH2^−^CD45^+^NKp44^−^CD117^−^CD127^+^ILC subset was altered differently in the inflamed terminal ileum of CD patients. They found that type 3 ILCs change their phenotype and function in inflamed terminal ileum. Compared with the normal terminal ileum, the frequency of NKp44^+^type 3 ILCs in CD patients was reduced, and the remaining type 3 ILCs secreted less IL-22 and produced only a small amount of IFN-γ. Interestingly, they also observed that NKp44^+^type 3 ILCs were negatively correlated with the enrichment of IL-17A^+^IFN-γ^+^ and IL-22^+^IFN-γ^+^T cells in the gut of CD patients. However, in uninfected tissue, NKp44^+^ILC3s produced only IL-22, not IL-17A or IFN-γ (27). These results confirmed that changes of intestinal NKp44^+^ type 3 ILCs in CD patients were directly correlated with changes of the IL-17A^+^IFN-γ^+^ or IL-22^+^IFN-γ^+^T cell subsets. Together, these results suggest that intestinal mucosal homeostasis is disrupted and the ILC phenotype is significantly altered in response to inflammation. Furthermore, the high expression of IFN-γ–producing type 1ILCs and decrease of type 3 ILCs in the intestinal tract of CD patients also demonstrated that ILCs are inextricably linked to CD (26, 28).

## Crohn’s Disease

CD is an inflammatory bowel disease (IBD) in which inflammation injures the intestines. It is a chronic condition, and so far, the mechanism is still unclear. It could be attributed to an imbalance of intestinal flora, or inheritance in the family. It is generally believed that CD is the result of interactions between genetic predisposition, environmental factors and changes in intestinal microbiota (26, 28). It can affect any part of the gastrointestinal tract from the mouth to the anus. CD usually occurs in young people, but can also affect children and the elderly (28). It is estimated that 500,000 people in the United States have IBD and that CD affects approximately seven out of 100,000 people (26). The annual incidence rate is 0–6 per 100,000 people and the prevalence is 1–5 per 100,000 in developing areas (29). Cohort studies from Asia, Africa and South America have consistently reported a rising incidence of CD in countries outside the Western world (30).

Epidemiological evidence strongly suggests that genetic factors are related to IBD susceptibility. Family studies reflect this convincingly, which shows that individuals with a family history have a much higher incidence of IBD than individuals without a family history (31, 32). In the twin studies, the rate of disease concordance in monozygotic twins was much higher than that of dizygotic twins (33). Furthermore, CD and UC, the two clinically similar disease share some genetic susceptibility loci (34, 35). In the past 15 years, great progress has been made in identifying further genetic risk factors for IBD. Genome-wide association studies have identified more than 200 IBD associated-susceptible genes, some of which are known to be involves or implicated in mediating host responses to gut microbiata (36). This has evoked the possibility that gut microbiota is implicated in the pathogenesis of IBD (37, 38).

The NOD2 gene was the first susceptibility gene for CD to be successfully identified in 2001 and Judy Cho et al. demonstrated the impact of NOD2 variants in European populations (34, 39). Interestingly, the finding indicated significant ethnic heterogeneity for researchers from China and Japan have not found association between NOD2 variants and CD in their populations and more further evidence (40–42). NOD2 is an innate immune receptor for muramyl dipeptide, which is a component of the bacterial cell wall. NOD2 gene product confers susceptibility to CD by altering the recognition of these components and/or by over-activating NF-κB in monocytes, thus documenting a molecular model for the pathogenic mechanism of CD (43, 44). Several genes have been studied so far with respect to CD, but thus far the strong and replicated associated have been identified with NOD2, IL23R and ATG16L1 genes (45). One mechanism which JAK2 contributes to CD pathogenesis could be by altering intestinal permeability. Prager et al. (46) showed that patients carrying the C risk allele within JAK2 rs10758669 displayed significantly more often an increased permeability compared with parents without the C allele (47).

Genetic susceptibility to CD is not limited to chromosome 16 and the recognition of a transduction pathway that, when dysregulated, contributes to the pathogenesis of CD will accelerate the discovery of additional susceptibility genes (48). In the past 50 years, the incidence of CD has dramatically increased in Western countries and more recently in the Asian-Pacific area, this suggests that environmental factors have an impact on the pathogenesis and incidence of CD, in addition to human genome coding relevant variant protein products.

**Figure 2:**
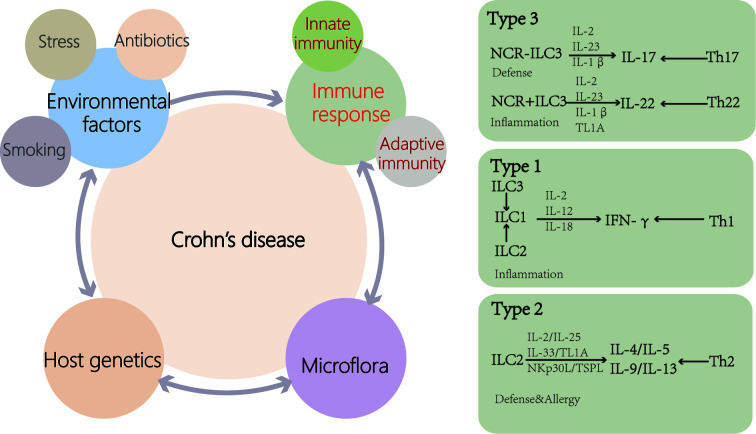
Causes of Crohn’s disease (CD). Genetic susceptibility is one of the important causes of CD patients. Disturbances of the microflora and environmental factors can cause abnormal immune responses, including innate and adaptive immunity, leading to intestinal inflammation. Disease outbreaks are related to environmental factors such as antibiotic use, stress and smoking. ILCs are activated by cytokines and cell surface-bound molecules to produce distinct sets of cytokines with important consequences for tissue homeostasis and disease. ILCs display a high degree of plasticity and can trans-differentiate depending on the inflammatory environment (TL1A, TNF-like ligand 1A).

Increased intestinal permeability leads to increased antigen exposure and activation of the innate immune system in CD (**Figure 2**). Compared with healthy people, the level of β-defensin in the ileum of CD patients is decreased (49, 50). Mutations in ATG16L have been found in CD patients and are associated with increased production of IL-1β (51), and IL-1β increases intestinal inflammation by inducing T helper (Th)-17 cells (52). According to past researches, the CD intestinal inflammatory infiltrate contains Th-1 and Th-17 cells. Choy et al. (28) suggested that ILC1 concentration in the ileum of CD patients was higher compared with that of the controls. These effector T-cell responses to bacteria or fungi are implicated in the pathogenesis of the disease. Impaired functional activity of intestinal Tregs cells has been reported in CD (53, 54). During CD, the epithelial barrier is breached as a primary or secondary event, and the luminal microflora stimulates a proinflammatory immune response by DCs and inflammatory macrophage. Regulatory ability of Treg is outstripped by inflammatory activity of Th1 and Th17. Additionally, ILCs, homoeostatic at steady state, contribute to the perpetuating inflammation, the production of cytokines (26).

The accumulation of IFN-γ–producing type 1 ILCs at the expense of IL-17/IL-22–producing type 3 ILCs in the inflamed intestinal tissues of CD patients. This plasticity of type 3 ILCs to type 1 ILCs may be one of the pathogenesis of CD. Recently, the result of a study investigated by Ying Tang et al. (55) suggest that STAT3 genotypic rs744166 and increased tyrosine phosphorylation of STAT3 in IL-23 responsive ILCs during pathogenesis of CD. They also found that IL-23–induced activation of STAT3 in the CD117^−^NKp44^−^ type1 ILCs involves in plasticity of type 3 ILCs to type 1 ILCs and a potential regulatory role of type 1 ILC function. In addition, it has been shown that STAT3 is essential for type 3 ILCs to produce IL-22 to protect intestinal tissues from infection in mice (56, 57).

Genes involved in CD regulate innate immune responses, bacterial killing, immune responses to endogenous microbial antigens and epithelial function. Different genetic abnormalities can lead to similar disease phenotypes. These genetic changes can be broadly characterized as causing defects in mucosal barrier function, immunoregulation or bacterial function. Chronic intestinal inflammation requires the presence of commensal enteric bacteria and activated T lymphocytes and patients with IBD and rodents with chronic intestinal inflammation exhibit loss of immunologic tolerance to normal enteric bacteria (58, 59). Although many IBD-associated dysbiosis have not been proven to be a cause or an effect of IBD, it is often hypothesized that at least some of alteration in microbiome is protective or causative (60). Overall, genetic susceptibility and innate immunity including ILCs, adaptive immunity, microbial flora, and environmental factors can be interrelated and combined, and contribute to the entire pathogenesis and development of CD.

## Accumulation of NKs and ILC1s

NK cells are a typical member of the ILC family and have extremely important anti-tumor and anti-infection activities because of their cytotoxicity and efficient production of IFN-γ. NK cells not only target tumor cells and malignant cells but also maintain the dynamic balance of the immune system by utilizing killing several types of activated immune cells (61).

NK cells are found throughout the human gut. They can be divided into CD56loNK and CD56hiNK types according to the differential expression of CD56 (62). NK cells expressing low levels of CD56 have stronger cytolytic activity. And those expressing high levels of CD56 are more sensitive to inflammatory cytokines and subsequently secrete cytokines, especially IFN-γ (7, 63, 64). Takayama et al. (65) found that the balance between NKp44^+^ and NKp46^+^NK cells was disrupted in CD patients. Moreover, translational evidence indicated that NKp46^+^NK cells were likely to mediate the pathogenesis of CD via IFN-γ. It can be produced by activated NK cells through IL-23. This is the result of an interaction between NKp46^+^NK cells and intestinal inflammatory macrophages (65).

There is substantial evidence that NK cells play a dual role in the intestinal tract, not only suppressing infection and inflammation but also promoting inflammation (61, 63, 66, 67). A mouse model showed that NK cells elicit IEC-related endoplasmic reticulum (ER) stress by increasing the expression of NKG2D ligands and making intestinal inflammation worse via recognizing and killing stressed intestinal cells through NKG2D (64). Cytolytic NK cells are enriched in the laminae propria of the colon in CD patients, and NK subsets generate inflammatory effects at the cellular level by producing pro-inflammatory cytokines that promote CD4^+^ T cell proliferation and CD-related Th17 differentiation (68, 69).

ILC1s are defined as CD127^+^T-bet^+^IFN-γ producing cells (5). They are unevenly distributed unevenly across parts of the human gastrointestinal tract and play a major role in the adult small intestine. There are two major type 1 ILC subsets in human intestine: lamina propria type 1 ILCs express CD 161 and CD 127, but not NKp44; epithelial type 1 ILCs express NKp44 and CD103 but lack CD 127, and their phenotype and function (in terms of cytotoxicity) are similar to that of classical NK cells.

Matthew et al. (28) reported that the number of type 1 ILCs in the ileum of CD patients was higher than that in the controls. CD127^−^type 1 ILCs are located in the intestinal epithelium, while CD127^+^type 1 ILCs are located in the lamina propria (70–72). The expression frequency of CD127^−^type 1 ILCs in inflamed tissue is higher than in non-inflamed tissue in CD patients. In addition, the number of CD127^+^ type 1 ILCs in the lamina propria also increases significantly. This is linked to the severity of the disease. Moreover, the increase of IFN-γ–producing type 1 ILCs is accompanied by a significant decrease in the number of NCR^+^type 3 ILC in the inflamed intestine (63, 71).

CD127^+^ lamina propria type 1ILCs and CD127^−^ epithelial type 1ILCs accumulate in the inflamed intestines of CD patients. This accumulation of type 1ILCs may be due to the ability of type 3ILCs to differentiate into type 1ILCs. Bernink et al. (71) observed that highly purified NKp44^+^type 3ILCs from fetal gut were able to differentiate into type 1ILCs in response to IL-2 and IL-12. This differentiation may be driven by the inflow of pro-inflammatory IL-12 phagocytosis at the infected site during intestinal inflammation. These findings indicate that a proportion of type 1 ILCs are derived from type 3 ILCs. Furthermore, data from a mouse experiment suggested that CD127^+^ type 1 ILCs can switch toward type 3 ILCs *in vivo* in the absence of inflammation (72). Type 1 ILCs do not exist in the intestines of the fetus, but they may be formed after the intestinal and symbiotic colonization of the intestine and become involved in early innate immune responses. Based on type 1 ILCs accumulation in the inflamed tissues of CD patients, they may be involved in the pathogenesis of the disease (73).

Bank et al. have made some breakthroughs through the mouse experiment. They identified c-FLIP as a target of cytokine-induced STAT5 activation, which is indispensable for the development of IL-15– and IL-17–dependent NKp46^+^ILCs (type 1ILC and type 1ILC) (74). c-FLIP is a master regulator of the extrinsic apoptosis pathway and protects immune and non-immune cells from caspase 8–dependent apoptosis (75, 76). c-FLIP–dependent NK46^+^conventional NK cells (cNK) protect T/B-sufficient mice from intestinal inflammation. The lack of cNK will limit the recruitment and pro-inflammatory functions of neutrophils, thus participating in the formation of colitis.

NK cells and type 1 ILCs intersperse in the intestinal lamina propria, and they share many of the same characteristics. They express transcription factor T-bet, can secrete IFN-γ and contribute to the cellular anti-bacterial process. These similarities often make them difficult to distinguish, and thus they are classified as one type of cells. As a matter of fact, NK cells and type 1 ILCs come from different sources. NK cells are derived from common innate lymphoid progenitor (CILP) via an NK cell precursor (NKP), whereas type 1 ILCs developing from CILPs via an ILC precursor (ILCP) (77). A significant difference between the two was previously thought to be that NK cells have cytotoxic effects. However, later research found that some type 1 ILC subgroups also mediate certain cytotoxic activities, although their effect may be minimal (NK cells and type 1 ILCs). NK cells and type 1 ILCs produce a large amount of IFN-γ that promote the migration of neutrophils, and activates lymphocytes, macrophages, and endothelial cells. In addition, IFN-γ affects the close connection function, resulting in damage to the epithelial barrier. In short, the production of a large amount of IFN-γ can exacerbate the occurrence and progression of inflammation.

## ILC2s in CD

In the definition of type 2 ILCs, they are GATA3^+^ and have the capacity of producing type 2 cytokines (e.g., IL-4, IL-5, and IL-13). Type 2 ILCs play an important role in enhancing the protective innate response to parasites and worms by secreting IL-5 and IL-13 to induce eosinophils and goblet cell proliferation and mucus production. This promotes the elimination of worms and prevents tissue damage (78). Although all type 2 ILCs apparently originate from the same precursor and secrete the type 2 cytokines IL-5 and IL-13, there may be some significant differences in surface markers, static transcriptional state and reaction to some stimuli in different type 2 ILCs in different tissues. This suggests that the function of type 2 ILCs can be affected by the organization environment, which, in turn, adapts to the organization’s specific steady-state requirements.

Type 2 ILCs exist at a lower frequency in intestinal ILCs. However, they also participate in maintaining gastrointestinal homeostasis and the progression of inflammation. It is reported that IL-33 can promote the secretion of amphiregulin by type 2 ILCs, which can act on the epidermal growth factor receptor (EGFR) on the epithelium and thus play a regulatory role to maintain intestinal epithelial homeostasis (79). Type 2 ILCs respond to stimulation of epigenetic cytokines such as IL-33, IL-25, and thymic stromal lymphopoietin, as well as eicosane cytokines such as prostaglandin D2 (PGD 2) and leukotriene D4 (80, 81). One prominent characteristic of activated type 2 ILC is secreting substantial amounts of IL-5, IL-9, IL-13, and amphiregulin (82). In addition to producing a series of cytokines and factors with unique functions, activated type 2 ILCs also regulates other cell types. For example, type 2 ILCs secret IL-13 to promote cup cell hyperplasia, thereby changing the intestinal epithelial barrier and inducing acidophilic cell proliferation by producing IL-5 and IL-9 (83). Like other ILCs, type 2 ILCs also play a dual role in intestinal homeostasis and the progression of inflammation (70, 84). Recent studies have shown that type 2 ILCs integrate pro- and anti-inflammatory signals, contributing to host defenses and epithelial barrier function. They also help organizational integrity and tissue-specific functions (83). Furthermore, type 2 ILCs also induce and amplify type 2 inflammation by increasing mucus production, eosinophils numbers, and Th2 differentiation once exposed to inflammatory stimuli. In addition to the aforementioned IL-33, IL-25, and thymic stromal lymphopoietin, other cytokines also play essential roles in regulating the type 2 ILCs response. IL-2 can not only directly induce the proliferation of type 2 ILCs but also acts as a common stimulation signal to promote type 2 ILCs to secrete cytokines. IL-4 promotes type 2 ILCs activation and responses in type 2 inflammation (85). Finally, production of type 2 cytokines and proliferation of type 2 ILCs also can be promoted via the IL-1R1 and NF-κB pathways through IL-1β signal transduction.

T-bet is not only essential for type 1 ILC and type 3 ILC biology, but also modulates type 2 ILC responses in the intestinal lamina propria. Selective loss of T-bet in ILCs leads to the expansion and increased activity of type 2 ILCs by the activation of GATA-3 related genes, which has a functionally important impact on mucosal immunity, including enhanced protection from Trichinella spiralis infection and inflammatory colitis (86). T-bet controls the intestinal ILC pool through regulation of IL-7 receptor signaling. The expression of T-bet in ILCs is a crucial transcription checkpoint for regulating pathogenic and protective mucosal immune responses, which is of great significance for understanding the pathogenesis of IBD and intestinal infection. Garrido-Mesa et al. reported that in a more physiologically relevant setting, in the presence of an otherwise intact immune system, T-bet deficiency in ILCs results in the development of protective mucosal immune responses (86). Previously, another article reported that T-bet expression in ILCs in the absence of an adaptive immune system works to dampen pro-inflammatory responses (87).

Forkel et al. (88) reported that type 1 ILCs increase in CD patients while type 2 ILCs increase in UC, especially early stage of the disease. Recently, it was reported that under stimulation with IL-12, type 2 ILC have plasticity to a type 1 ILC cytokine profile (89). Furthermore, some certain type 2 ILCs in the mucosa of CD patients can produce IFN-γ in addition to its symbolic cytokine IL-13 (89). This shows the manifestation of type 2 ILC plasticity in CD (90). In an inflammatory environment, the type 2 ILCs phenotype changes so dramatically that cells likely to adopt some or all of the typical type 1 ILCs or type 3 ILCs transcription profiles (83). Investigators have also shown that type 2 ILCs can be converted into type 3 ILC-like type 2 ILCs and express IL-17 along with type 1 ILC-like type 2 ILCs and IL-13. Thus, these unique type 2 ILCs acquire type 1 ILC-like features, including IFN-γ production and expression of T-bet, while having lower expression of GATA3 and ST2 (89).

The study of Lim et al. (89) confirmed that the induction mechanism of type 2 ILC plasticity mapped to the IL-12-IL-12R signaling pathway. In addition, the authors detected IL-13^+^IFN^+^type 2 ILC precursors in intestinal samples from patients with CD. The plasticity of type 2 ILCs and type 1 ILCs is bidirectional: type 2 ILC-derived type 1 ILCs can revert to type 2 ILCs via regulation of IL-4. *In vitro*, cytokines IL-12 and IL-18 can induce type 2 ILCs to differentiate into type 1 ILCs. Transferred type 2 ILCs seem to affect infected mice through partial conversion to type 1 ILC-like type 2 ILCs. Furthermore, type 2 ILCs appeared to show a potential role in tissue fibrosis (91). However, whether the increased frequency of type 2 ILCs is related to the development of fibrosis and stenosis in CD patients is still unclear (88).

## Plasticity of ILC3s in CD

Type 3 ILCs are the largest ILC group involved in human intestinal stability (90). Based on the expression of T-bet and CCR6, two different type 3 ILC subgroups can be distinguished, and they both express RORγt (92). The characteristic cytokines of IL-3 are IL-22 and IL-17. Interestingly, Geremia (81) have shown that the composition of different ILC subsets is related to local IL-7 level. That means IL-7 can regulate the subsets of ILCs in the human body, and more IL-7 can lead to more type 3 ILCs (25). In the healthy intestinal tract, type 3 ILCs exhibit a range of cytokine-dependent and cell-surface receptor-mediated mechanisms that participate in intestinal immunity and homeostasis maintenance. Type 3 ILCs maintains a balance and barrier integrity between the enhancement and suppression of the immune response against the microbiome (93).

Type 3 ILCs can maintain intestinal homeostasis through the following three mechanisms: 1) By receiving the signal IL-1β signal of macrophages through surface receptors, type 3 ILCs secrete granulocyte-macrophage colony-stimulating factor (GM-CSF), which regulates dendritic cells (DCs), macrophages and various cytokines, thus controlling the number of regulatory T cells and maintaining intestinal homeostasis (94); 2). Type 3 ILCs secrete IL-22 and lymphotoxin, which induce IECs to express fucosaccharide transferase to promote fucosaccharide glycosylation for maintaining intestinal homeostasis (95); and 3) By expressing major histocompatibility complex-II (MHCH II) molecules, MHCH^+^type 3 ILCs can directly induce T cell death targeting intestinal symbiotic bacteria.

Type 3 ILCs can maintain intestinal homeostasis by directly regulating and interacting with other immune cells. IL-23 and IL-1β stimulate type 3 ILCs to produce the effector cytokines, IL-22, GM-CSF, and IL-17, when bacteria invade. Among type 3 ILC products, as a crucial cytokine, IL-22 also can be secreted by Th17 and Th22 cells and plays a key role in controlling homeostasis control (92, 96, 97). IL-23 is a key activator of type 3 ILCs that drives colitis. Moreover, Type 3 ILCs regulate the adaptive responses of Th17 cells (77, 95). Foxp3^+^ regulatory T cells (Tregs) prevent type 3 ILC-mediated colitis by inhibited IL-23 and IL-1β production from intestinal-resident CX3CR1^+^ macrophages. Moreover, Tregs restrain type 3 ILC production of IL-22 through suppression of CX3CR1^+^ macrophages production of IL-23 and IL-1β (98). The secretory response of type 3 ILCs to IL-1 and GM-CSF is a key regulator of intestinal regulatory T cells induced by mononuclear macrophages. Furthermore, stimulation with IL-1β induces the production of pro-inflammatory cytokines in type 3 ILCs, including tumor necrosis factor (TNF)-α, IL-6, and GM-CSF, and regulates the expression of MHCII and synergistic stimulation ligands. A recent study found that microbiota-induced TNF-like ligand 1A (TL1A) is able to drive type 3 ILC-mediated barrier protection and intestine T cell activation during colitis. Using cell-specific genetic deletion models, Castellanos et al. help to revealing a central role for type 3 ILC in mediating acute protection in acute colitis models, but promoting DR3-dependent pathogenic T cell responses in chronic disease (99–101). In IBD patients with genetic susceptibility or animal models of T cell transfer colitis, type 3 ILC co-stimulation can support pathogenic inflammatory T cell activation. During acute colitis, TL1A synergy with IL-23 promotes mucosal healing, but sustained intestinal damage leads to the up-regulation of OX40L on type 3 ILC and co-stimulation of inflammatory intestinal T cells.

RORγt type 3 ILCs are involved in the induction of inflammation and the pathogenesis of some autoimmune diseases, including CD and ulcerative colitis. Unlike ulcerative colitis, CD patients have an accumulation of LIN-CD56-CD127ILCs in the inflamed ileum and colon (25). Type 3 ILCs accumulate in the intestinal tract and are induced by IL-23 to produce IFN-γ. On the other hand, type 3 ILCs accumulate in the lamina propria and are activated by tumor necrosis factor-α, IL-23, and IL-6 produced by activated DCs. By gathering and moving into crypts, ILCs, including type 3 ILCs, may initiate inflammatory immune cascades that lead to intestinal inflammation. Mobilization of type 3 ILCs out of the crypts may play a role in coordination and perpetuation of the inflammation in the gut (102). Final reduction of type 3 ILCs can result in contraction of the inflammation. However, research by Li et al. shows that blocking loss of type 3 ILCs may also simultaneously ameliorate colitis by limiting the pathogenicity of type 3 ILCs or other mechanisms (102–104). They have found that activation of DR3 signaling leads to exacerbation of the colitis through targeting type 3 ILCs, which were finally eliminated from the intestine through GM-CSF/IL-23–dependent mechanism (103). Therefore, blockade of DR3 using antibodies resulted in ameliorated colitis likely by suppressing the pathogenicity of type 3 ILCs. However, it is difficult to assess the consequence of type 3 ILCs *per se*, without affecting cytokine production of type 3 ILCs, and the effect of abnormal retention of pro-inflammatory type 3 ILCs will be one of the important issues in future research.

Consumption of ILCs leads to infection by commensal bacteria and systemic inflammation, and these events can be suppressed by modulation of IL-22. RORγt^+^ type 3 ILCs and IL-22 secretion play vital roles in intercommunication among cells (105, 106). IL-22 produced by type 3 ILCs binds to IL-22R to trigger the generation and maintenance of IL-18 mRNA and induce IL-18 signal cascades in epithelial cells. Therefore, the production of IL-18 and IL-22 is closely and mutually controlled. In the mouse studyby Munoz et al. (107), IL-22 induces mice to produce IL-18 through IECs, while uncontrolled IL-18 amplifies intestinal inflammation, which destroys the mucosal barrier of mice and causes intestinal inflammation. In intestinal lesions and peripheral blood of CD patients, IL-18 is up-regulated (108), indicating that the regulation of IL-22 and IL-18 is changed. Moreover, type 3 ILCs are also found in mice and humans and secrete GM-CSF. The GM-CSF producing type 3 ILCs can absorb myeloid cells and further promote intestinal inflammation. Activated type 3 ILCs move through the intestinal tissue, and promote inflammation and development throughout the entire intestine tract (102).

Loss-of-function studies defined that type 3 ILC-derived IL-2 is essential to maintain regulatory T cells (Tregs), immunologic homeostasis and oral tolerance to dietary antigens uniquely in the small intestine (109). IL-2 is a multidirectional cytokine necessary for the prevention of chronic inflammation of the gastrointestinal tract. The protective effects of IL-2 involve the production, maintenance and function of Tregs (110–112). Low dose of IL-2 has become a potential treatment strategy for patients with IBD (113). IL-1β selectively induces IL-2 transcription and protein production in type 3 ILC, which is mainly produced by macrophages. Type 3 ILC-derived IL-2 is a novel and non-redundant pathway that supports the population size and homeostasis of peripherally-induced Tregs in the small intestine (109), and further that this pathway becomes dysregulated in the context of CD. Impaired type 3 ILC-derived IL-2 is linked to a reduction of Tregs and impaired immune regulation within the small intestine of CD patients.

In the patients with CD, type 3 ILCs produced an increased amount of IL-17A. At the same time, some type 3 ILCs may be transformed into type 1 ILCs, leading to an increase in the proportion of type 1ILCs. Furthermore, the number of MHCII^+^ type 3 ILCs decreases but Th17 cells increase in CD patients, and changes in the number of these cells will eventually lead to intestinal inflammation (92). Type 3 ILCs have been shown to differentiate into type 1 ILCs from IL-12 produced by CD14^+^DCs. Type 3 ILCs reduced the expression of RORγt, and subsequently express T-bet, NK1.1, and NKp46, and secrete IFN-γ, which gradually causes the acquisition of type 1 ILC characteristics. This may be a reason why patients with CD have an abnormal balance of type 1 ILCs. However, this differentiation is reversible; DCs guide ILC plasticity, and different DC subgroups induce type 1 ILCs to differentiate into type 3 ILCs. The synergy between IL-23 and CD14^−^DC–produced IL-1 and vitalic acid can cause these type 1 ILCs to redivide into type 3 ILCs (72). Moreover, researchers have identified that the differentiation toward type 1 ILCs was driven by IL-12, and conversely, IL-23 promoted polarization toward type 3 ILCs *in vitro* and *in vivo* (114). Ying Tang et al. indicated that increased STAT3 activity was associated with the pathogenesis and progression of CD (The expression of STAT3 mRNA, STAT3-related genes, and activation of the STAT3 signal (phosphorylated STAT3) were increased in the ileum in CD patients). Particularly, activated STAT3 signaling in IL-23 responsive ILCs may lead to the chronic relapse of CD (55). Further study on the gene regulation of STAT3 in ILCs will help to further explore the exact molecular mechanisms of ILC plasticity and heterogeneity.

## Conclusions

**Figure 3:**
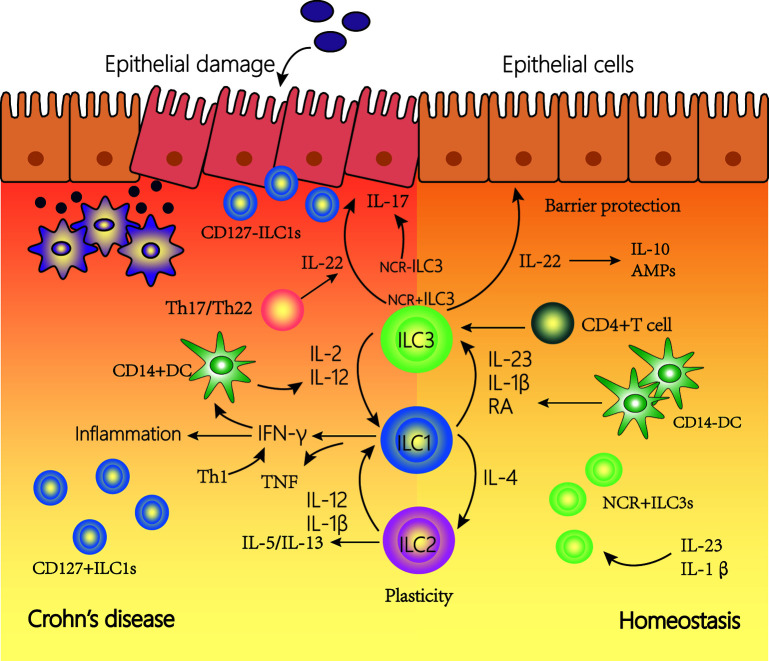
The plasticity of innate lymphoid cells (ILCs) contributes to Crohn’s disease (CD). In a healthy state, NCR^+^type3 ILC form the majority of ILCs in the lamina propria of the intestine. IL-22 is then produced, which is important for tissue regeneration and the secretion of mucous, antimicrobial peptides (AMPs) and IL-10. In the inflamed intestine of CD patients, the majority of ILCs has a type1 ILC phenotype and produce IFN-γ and TNF. The plasticity of type 2 ILCs and type 3 ILCs, allowing trans-differentiation into type 1 ILCs involved in the pathogenesis of intestinal inflammatory diseases such as CD. Under the influence of IL-12 and IL-2 regulated by DCs, the differentiation of type 3 ILCs to type 1 ILCs increases. In addition, under the action of IL-12, type 2 ILCs can also be converted into type 1 ILCs. Type 1 ILCs, before and after transformation, jointly promoted the increase of interferon (IFN)-γ production. This transformation is reversible, and type 1 ILCs can revert to type 3 ILCs in the presence of IL-23, IL-1β, and retinoic acid (RA) secreted by DCs.

Three ILC subsets play a crucial role in barrier function and innate immune defense (89, 95, 115). Type 1 ILCs are rich in the upper digestive tract, while type 2 ILCs appear only at very low frequency throughout the intestines and type 3 ILCs appear at a high frequency (88). The roles of ILCs in function of the gut manifest functions in two ways. First, ILCs play a protective role in the intestinal tract by regulating the microbial niche and contributing to epithelial and tissue repair. Second, ILCs induce inflammation by producing inflammatory cytokines and regulating natural and adaptive immune responses. ILC can function not only by secreting soluble factors but also by interacting with other cells such as immune cells. ILCs play an important amplifying role in CD via the production of inflammatory cytokines production and interactions with other immune and non-immune cells (**Figure 3**). In CD patients, there is an accumulation of IFN-γ–secreting ILCs in both the lamina propria and the epithelium. In addition, through the connection of ILCs, the intestinal flora and ENS can play an important role in the gut. ILCs can secret inflammatory cytokines such as IL-22 induced by neurons and ECGs. The gut microbiota interacts with some cells (such as T cells and IECs) to induce or inhibit ILCs to secrete inflammatory cytokines. Additionally, the release of these cytokines can also change the intestinal flora (12). ILCs may link the intestinal flora and ENS and work together in the intestine (116, 117).

ILC subgroups can be inter-converted. This may be one reason why different subgroups change in frequency during the progression of intestinal inflammation. The plasticity of ILCs may support a fast, flexible immune response that promotes tissue integrity, but can also lead to changes in tissue stability, leading to persistent organ dysfunction. This may contribute to the development of CD.

Over the past decade or so, scientists have gained a deeper understanding of the classification of ILCs, and their functions and developmental differentiation process (118). However, the understanding and discovery of ILCs are only the “tip of the iceberg”, and many dilemmas remain to be resolved. The classification and division of some ILC subgroups are still unclear because there is no appropriate method to identify the different subgroups that are highly similar, such as type 1 ILC and NK cells (119). If the cell-specific surface markers of each ILC subgroup can be recognized and selectively labeled, it will be helpful to find a new treatment for CD. However, if using ILCs as a target therapy to help heal damaged intestinal mucosa and resist infection, will it disturb the mucosal homeostasis in other parts of the patients? This requires more experimentation and data for further study.

It was mentioned previously in this article that type 3 ILCs in inflamed tissue in CD patients can be differentiated to type 1 ILCs. However, this ability only appeared in the inflammatory site. Although type 1 ILCs were also differentiated to type 3 ILCs in normal tissue environments in mouse experiments, further studies are required to determine whether this is the case in the human body. The specific effect and mechanism of abnormal retention of pro-inflammatory type 3 ILCs on IBD are still worth exploring. Furthermore, some investigators have suggested that ILCs play a unique redundant role for redundancy with T-cells. Thus, in the pathophysiology of inflammatory diseases, it is often difficult to distinguish between the roles of ILC and T cells.

In conclusion, although it has been shown that the changes in ILCs phenotypes and functions in the human intestine under chronic inflammatory conditions are inextricably linked to the development of CD, many specific mechanisms are still unclear. More in-depth analyses and research into the ILC subgroup and the connection between the microbiota, adaptive immune cells in the future may lead to a deeper understanding of the role of these cells in CD. As part of the first line of defense in the intestines when inflammation occurs, ILCs may also be part of the pathogenesis of CD. Thus, research on ILCs can provide new insights into the treatment strategies of CD patients by understanding and studying the production and function of these disease-causing cells.

## Author Contributions

YW collected the papers and data, analyzed the conclusions, and drafted the manuscript. JS presented the idea of this paper, supported the funding, analyzed the conclusions, drafted, and revised the manuscript. All authors contributed to the article and approved the submitted version.

## Funding

Supported by grants from National Natural Science Foundation of China (No. 81770545) and MDT Project of Clinical Research Innovation Foundation, Renji Hospital, School of Medicine, Shanghai Jiaotong University (PYI-17-003).

## Conflict of Interest

The authors declare that the research was conducted in the absence of any commercial or financial relationships that could be construed as a potential conflict of interest.
